# Synchrotron-Based in Situ Characterization of the Scaffold Mass Loss from Erosion Degradation

**DOI:** 10.3390/jfb7030017

**Published:** 2016-07-05

**Authors:** Nahshon K. Bawolin, Xiongbaio Chen

**Affiliations:** 1 Department of Mechanical Engineering, University of Saskatchewan, Saskatoon, SK S7N 5A9, Canada; nkb845@mail.usask.ca; 2 Division of Biomedical Engineering, University of Saskatchewan, Saskatoon, SK S7N 5A9, Canada

**Keywords:** erosion degradation, synchrotron-based imaging, tissue scaffolds, mass loss

## Abstract

The mass loss behavior of degradable tissue scaffolds is critical to their lifespan and other degradation-related properties including mechanical strength and mass transport characteristics. This paper presents a novel method based on synchrotron imaging to characterize the scaffold mass loss from erosion degradation in situ, or without the need of extracting scaffolds once implanted. Specifically, the surface-eroding degradation of scaffolds in a degrading medium was monitored in situ by synchrotron-based imaging; and the time-dependent geometry of scaffolds captured by images was then employed to estimate their mass loss with time, based on the mathematical model that was adopted from the literature of surface erosion with the experimentally-identified model parameters. Acceptable agreement between experimental results and model predictions was observed for scaffolds in a cylindrical shape, made from poly(lactic-co-glycolic) acid (PLGA) and polycaprolactone (PCL). This study illustrates that geometry evaluation by synchrotron-based imaging is an effective means to in situ characterize the scaffold mass loss as well as possibly other degradation-related properties.

## 1. Introduction

**Figure 1:**
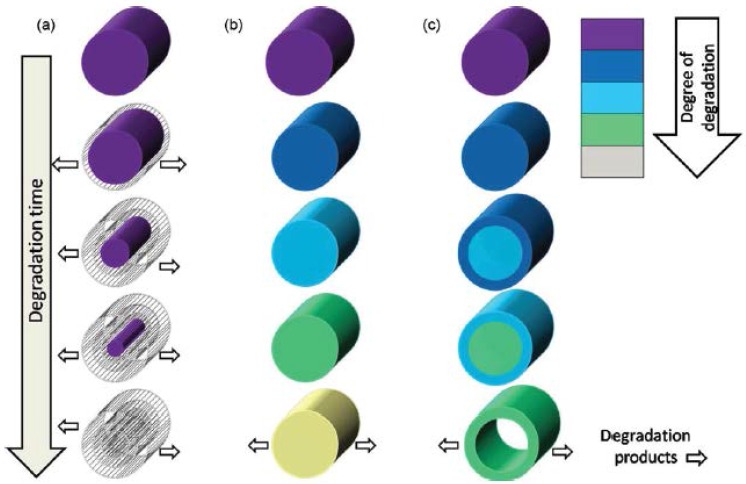
(**a**) Surface degradation; (**b**) homogeneous bulk degradation; and (**c**) inhomogeneous bulk degradation [3].

Tissue engineering is an emerging field with one goal of developing methods to replace/repair damaged and/or diseased tissue. One of the most promising state-of-the-art methods for creating new tissue is scaffold-based tissue engineering. In this method, an artificial matrix—known as a tissue scaffold—is seeded with patient derived cells, forming what is known as a tissue engineering construct. This construct is then either directly implanted within the body, or incubated within an artificial simulacrum of the living environment for a time period prior to its implantation. The cells seeded within the scaffold self-organize into new tissue/organs, while the underlying scaffold degrades and is usually absorbed and excreted by the body. A critical factor in the design of the tissue scaffold is its degradation behavior, which controls the device’s lifespan in the body and other related features such as its mechanical strength [1,2] and mass transport characteristics, which are both functions of time. Synthetic and natural polymers display two general types of degradation behavior, i.e., (1) surface degradation, as illustrated in Figure 1a; and (2) bulk degradation. Within the category of bulk degradation there are two additional degradation pathways; (1) uniform degradation and (2) heterogeneous degradation [3], which are illustrated in Figure 1b,c, respectively. Examples in the literature of scaffolds that display surface degradation are found in references [4,5,6], while examples of bulk degrading scaffolds are in [7,8,9] and inhomogeneous bulk degradation is in [10].

**Figure 2:**
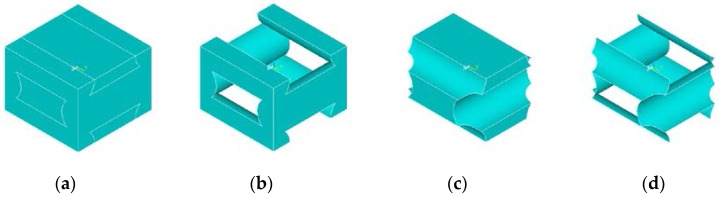
(**a**) Tissue engineering construct unit cell; (**b**) scaffold material occupied volume; (**c**) tissue/cells/fluid occupied volume; and (**d**) interface surface between the two volumes.

Part of the volume of the tissue engineering construct is occupied by the tissue scaffold material phase, and the remaining volume is filled by a fluid, seeded cells, and developing tissue. Figure 2a shows an example of a unit cell of a tissue engineering scaffold, while Figure 2b–d illustrate the scaffold material occupied volume in the unit cell, the tissue/cells/fluid occupied volume, and the interface surface between these two volumes, respectively.

In surface degradation, the scaffold material resists the infiltration of the liquid degrading medium, resulting in a steep gradient of medium concentration within the scaffold material. The maximum concentrations occur near the interface between the scaffold-material volume and the tissue/cells/fluid volume and then decreases toward the inside of the scaffold material. As such, the degradation chemical reaction (hydrolysis) proceeds at a higher rate at the interface surface than in the interior of the scaffold-material volume, which further causes the molecular weight of the scaffold material to decline fastest near the interface surface. Once the molecular weight decreases to a critical level, the polymer chains diffuse from the scaffold into the fluid/cell/tissue volume. This erosion process changes the scaffold geometry, however it does not alter much of the molecular weight—and therefore the mechanical properties—of the scaffold-material volume. In essence, small sheet like layers of the scaffold material are dissolved and stripped away, revealing a fresh surface which then in turn is attacked by the degrading medium. Over time, the scaffold-material volume decreases in size as the scaffold material is gradually dissolved, and the in-solution scaffold materials are disposed by the body. 

The degradation behavior of tissue scaffolds is usually experimentally characterized by in vitro tabletop degradation experiments, where a tissue scaffold sample is immersed in phosphate-buffered saline (PBS) and incubated at body temperature [11]. While these experiments provide some useful data about degradation, the PBS medium does not perfectly represent in vivo degradation. To achieve closer agreement between in vivo and in vitro degradation experiments, media that includes enzymes and cultured cells have also been employed in degradation testing [12,13]. It is not yet practical or cost effective to perfectly replicate the living environment in vitro by representing every biological and chemical effect found in the body artificially [14].

The differences between in vivo degradation and in vitro degradation in PBS can be eliminated by animal testing [15,16,17,18]. This approach requires a large number of animals to evaluate even a small sample size, and the analysis of explants can also only measure the average properties of the entire extracted device; i.e., location specific properties are not measurable. This limitation can be overcome by adopting image-based methods to evaluate scaffold degradation and mass loss [19]. However, even if imaging-based methods are utilized to characterize surface degradation induced sample mass loss, ex vivo imaging methods still require the removal of the implant from the animal [20,21,22], which results in some variability from the act of removing the sample and the variation between animals. In [23] the changing density and morphology of bone is evaluated in vivo by X-ray micro computed tomography (µ-CT) scans, raising the possibility that a similar procedure may allow the same type of experimental evaluation to be performed for tissue scaffolds. Unfortunately, this absorption-based method has difficulty distinguishing soft tissue from the background, and is only viable for bone tissue engineering since the X-ray absorption characteristics of bone provide sufficient contrast with the soft tissue surroundings. This limitation may be overcome by in vivo/in situ image-based methods that can provide sufficient contrast to visualize the low density polymeric scaffold or devices against a tissue background with similar X-ray attenuation properties [24,25,26,27,28,29]. 

**Figure 3:**
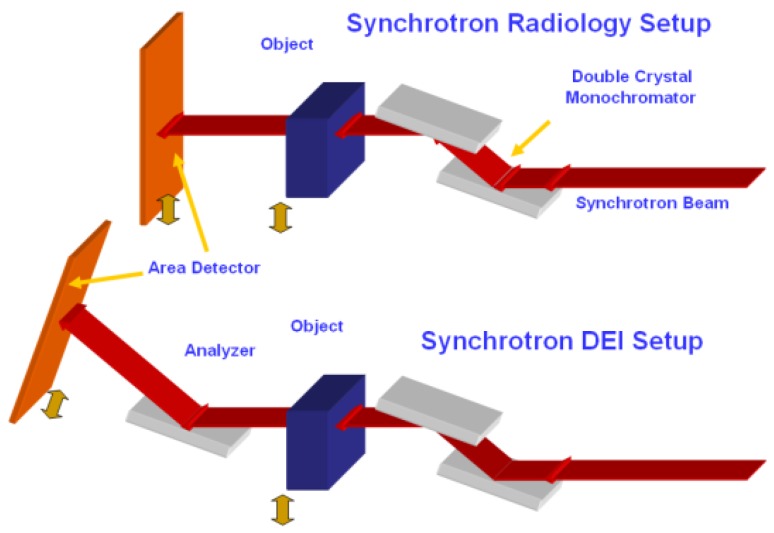
Diffraction-enhanced-imaging (DEI) and conventional radiography beamline experimental setups.

Diffraction-enhanced-imaging (DEI) is a well-developed imaging method that shows promise as a way to monitor implants when they are surrounded by material with similar densities and absorption properties in vivo. The beam-line configuration of the DEI method is illustrated in Figure 3, as compared to that of conventional radiography synchrotron based absorption X-ray imaging. The major difference between them is the use of analyzer, where only the part of the X-ray with a small scattering angle is selected and diffracted into the detector in the DEI, with more details available in [30,31,32,33].

In this study, we hypothesize that synchrotron-based DEI can be used to characterize the geometry of implants and then to infer their mass loss, as they erode with time. To test it, we proposed to employ computed tomography to reconstruct the scaffold geometry with time, which allows for the characterization of the volume of remaining scaffold material at multiple time points during its degradation. On this basis, we should be able to determine the mass loss with the known density of this scaffold material. It should be noted that while theoretically, it should be possible to detect the porosity that develops in bulk degrading polymers [34], the resolution requirements for this task are not currently available and thus this method is presently limited to the consideration of surface degrading tissue scaffolds only. 

The approach proposed above, while possible, possesses unresolved issues. In living biological samples, the allowable radiation dose would restrict the number of time points for imaging, thus limiting the information for inferring the mass loss over of scaffold the course of its degradation. Computed tomography imaging also requires a significant amount of time, in an order of hours (especially if high resolution is required [35]), which is problematic in situations where the degradation rate of scaffolds is too fast to be captured by imaging. In this work, planar images of the outer surface of the scaffold are employed to determine the average strand diameter of the scaffold with time, and the degradation is then assumed to be uniform throughout the scaffold. The change in strand diameter is employed to estimate the volume of the scaffold with time, and its mass at a specific time is then inferred from this volume and the known density of the scaffold material.

## 2. Experiments and Methods

The polymer polycaprolactone (PCL) was chosen as the material employed for rapid prototyping scaffolds, and the degradation medium was chosen to be a 5M base solution of sodium hydroxide at a temperature of 55 °C. Sodium hydroxide is chosen as the degradation medium because sodium hydroxide ions do not diffuse readily into a wide range of polymer materials, which results in surface degradation behavior [36,37]. The degradation of devices is also significantly accelerated compared to that experienced in phosphate-buffered saline. This particular selection of degradation medium and material can act as a demonstrative system to represent a variety of biologically relevant and general polymer analysis combinations of material and degradation media. To represent an implant with different geometry manufactured out of a different material degraded by a less aggressive degradation medium, poly(lactic-co-glycolic) acid (PLGA) cylinders were manufactured and degraded as well in a 1 M solution of sodium hydroxide at 55 °C. In detail, PLGA 50:50 with a viscosity based molecular weight of 100,000 Daltons was purchased from Lactel Corporation (Birmingham, AL, USA). The polymer was raised to an appropriate temperature in a furnace and cast into cylindrical Teflon molds 1 cm high and 0.8 cm in diameter. The samples were removed from their molds with a metal punch and the surfaces of the plastic cylinders were machined via lathe to achieve consistent sample geometry. Twenty-four samples of PLGA were produced. 

Polycaprolactone with an initial number average molecular weight of 80,000 Daltons was purchased from Sigma Aldrich (Munich, Germany) and dispensed into three dimensional tissue scaffolds with an Envisontech bioplotter. The polymer was extruded as a melt at 200 °C into strands of 500 µm diameter spaced 800 µm apart. Each layer of strands was dispensed 700 µm above the previous layer. These dimensions were verified by optical microscopy, where dimensions were measured with a microscope camera and the software Paxit. The overall dimensions of each scaffold were 5 mm × 5 mm × 5 mm.

The sample initial weights were measured and the PLGA and PCL samples were immersed in solutions of NaOH and incubated at 55 °C. The PLGA implants were immersed in a 1 Molar (M) solution while the PCL samples were degraded in a 5 M solution. Preliminary experiments [38] revealed that these highly basic mediums and elevated temperatures induced accelerated degradation and induced surface degradation in the PLGA and PCL samples. The PLGA samples were degraded for 54 h with samples removed at *t* = 0, 12, 24, 30, 36, 42, 48, and 54 h. For PCL, the scaffolds were removed and characterized at *t* = 0, 5, 20, 25, 30, 35, and 45 h. All samples were then reweighed and placed in a 2 cm thick ethanol/water filled tank and imaged at the Canadian Light Source (CLS) Biomedical Imaging and Therapy bend-magnet beam-line (BMIT) at an energy of 40 keV. Three images of each sample were taken; one at the peak of the rocking curve and two at opposite sides of the rocking curve at half intensity. This particular beamline is equipped with a (2,2,0) cut silicon monochromator and analyzer crystal. A copper filter was applied to remove the (2,2,0) reflection leaving only the (4,4,0) reflection at 40 keV to proceed through the sample. The diameter of the deposited strands that make up the layers of the rapid prototyping manufactured scaffold were measured at 20 separate locations on the scaffold, and these measurements were employed to estimate the remaining volume of the scaffold at each time point. 

The semicrystalline PCL samples were not expected to experience crystallization during their degradation in NaOH, since degradation is confined to the interface surface of the scaffolds. But differential scanning calorimetry (DSC) was still performed to ensure that the PCL crystallinity had not changed with time during degradation. The DSC method melts samples of the scaffold and evaluates the heat absorbed by the scaffold during its phase change, which is the conversion of its ordered crystalline phase into a fully amorphous microstructure. By comparing the enthalpy required to transform the samples to their fully amorphous state to that required for a fully crystalline sample, the crystallinity of the scaffold may be obtained [39]. The treatment of the samples involved heating from room temperature to 100 °C at a heating rate of 10 °C/min and a return to room temperature after stabilization. The phase change initiated at a transition temperature of 60 °C which is typical for PCL.

## 3. Modelling

To represent the scaffold mass loss, the phenomenological model reported in the previous study [40] was adopted with the parameters to be identified by means of experimental date captured by the DEI images. In this model, a hydrolysis reaction reduces the molecular weight of a degrading polymer scaffold, denoted by $\dot{R}$, which represents the number of polymer chains cut per interval of time.

(1) $$\dot{R}=-dC_{e}/dt=k_{1}C_{e}C_{w}+k_{2}C_{e}C_{w}C_{m}{}^{0.5}+k_{3}C_{e}C_{w}C_{m}C_{\text{NaOH}}{}^{0.63}$$

$$M_{w}/M_{w0}=C_{e}/C_{e0}$$

This rate of chain destruction is dependent upon the concentration of ester bonds *C_e_*, the concentration of water *C_w_*, and the concentration of any media that may change the local pH of the surroundings. These may include the acidic byproducts of degradation *C_m_* or some substance present in the surrounding medium, such as basic sodium hydroxide *C*_NaOH_. It may also include the byproducts of metabolism generated by the cells seeded in the scaffold. In the particular degradation conditions selected for this study, the degradation rate from the presence of water and weak acidic degradation byproducts is much less pronounced than that induced by the presence of the sodium hydroxide. Therefore, the simplified model of degradation may only consider this chemical species and its mass transport inside the implant. The hydrolysis rate parameters for water and monomers *k*_1_ and *k*_2_ may be ignored leaving only *k*_3_ as an unknown parameter in the degradation model that must be estimated from the experimental data. The terms above the concentration of monomers and sodium hydroxide are dissociation terms, relating concentration to the amount of the species that dissociates in solution. The diffusion coefficient of the species within the implant increases with time due to the increase in the porosity of the material. The porosity of the polymer is approximated by,

(2) $$\rho =1-C_{e}/C_{e0}$$

and the effective diffusion coefficient of the degrading polymer is estimated by the Maxwell Garnett expression for the effective properties of a mixture, when the inclusions of the second phase in the mixture are spherical [41].

(3) $$D_{ap}=D_{a}\left[1+\frac{3\left(D_{p}-D_{a}\right)\rho }{D_{p}+2D_{a}-(D_{p}-D_{a})\rho }\right]$$

where *D_p_* is the diffusion coefficient in the pores, *D_a_* the amorphous phase of the material, and ρ the time dependent porosity of the amorphous polymer phase. At a critical molecular weight, the polymer is assumed to become soluble in the degrading medium and diffuse out of the implant. For PLGA this critical molecular weight has been experimentally determined to be 15,000 Daltons [42]. For PCL this critical molecular weight is 5000 Daltons [43]. In each element of the model, the scission rate is calculated, and a random parameter is assigned as the fraction of scissions that have produced polymer chains less than the critical molecular weight in size. The mass loss in each element is then calculated as,

(4) $$\frac{M_{t}}{M_{0}}=\frac{N_{t}}{N_{0}}$$

where *M_t_* is the mass at time *t*, *M*_0_ is the initial mass of the element, and *N_t_* and *N*_0_ are the number of polymer chains in each element with molecular weights greater than the critical molecular weight respectively. For PLGA, the diffusion coefficient of NaOH was chosen to be similar to that of a carboxyl monomer in an amorphous polymer; 3.6 × 10^−7^ cm^2^/h. For PCL, the diffusion coefficient of NaOH was chosen to be higher, since the PCL displayed bulk degradation. A value of 8.29 × 10^−4^ cm^2^/h was chosen, which is equal to the measured diffusion coefficient of water in PCL. In order to estimate the unknown parameter in the model, an objective function was defined and minimized.

(5) $$Obj(k_{3})=\sum_{t=0}^{t=k}abs(M_{\exp}(t)-M_{\mathrm{mod}el}(t))$$

where *t* is degradation time, *k* is the time at the end of sample degradation, *M*_exp_(*t*) is the experimentally measured mass loss as a function of time, and *M*_model_(*t*) is the time dependent model prediction for mass loss.

## 4. Results and Discussion

**Figure 4:**
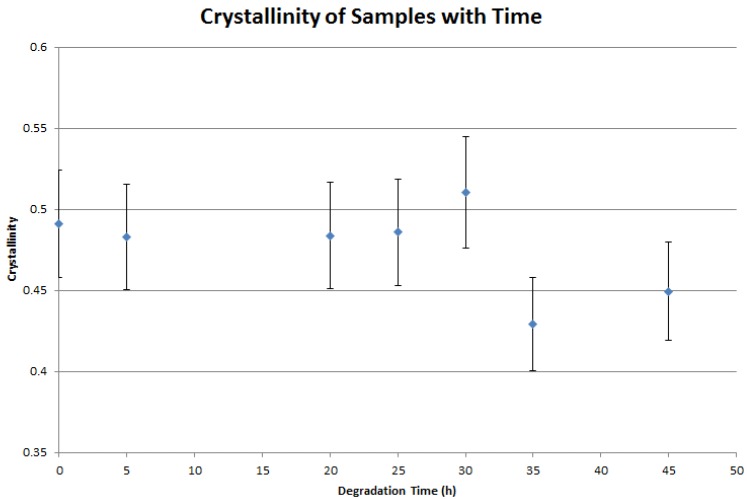
Crystallinity of polycaprolactone (PCL) scaffolds with time.

The crystallinity of degrading semicrystalline polymeric samples has been observed to gradually increase with time during degradation [44]. While degradation is confined to the interface surface of the scaffold, there is a possibility that a thin layer of partially degraded material may exhibit changes in crystallinity during degradation. Furthermore, the elevated temperature of the degradation medium may also lead to an increase in sample crystallinity. Since density is related to the crystallinity of the sample, a change in this quality may yield a change in geometry that is not related to mass loss, which would lead to an overestimation of mass loss from the characterization of the scaffold geometry. Also, it may be of interest to extend this work in the future to estimate mechanical properties from geometry, and such estimations would be complicated by mechanical property changes that were not a function of geometry. Monitoring of PCL scaffold crystallinity yielded the following results, illustrated in Figure 4, which indicate that there is no appreciable crystallization of the sample during surface degradation.

**Figure 5:**
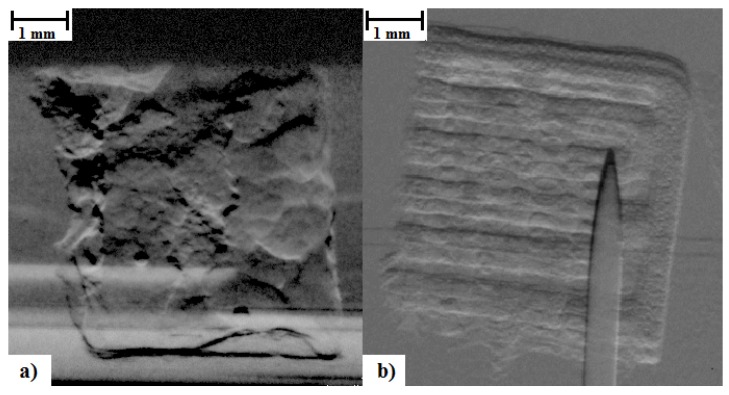
DEI image of (**a**) poly(lactic-co-glycolic) acid (PLGA) cylinder and (**b**) PCL tissue scaffold.

**Figure 6:**
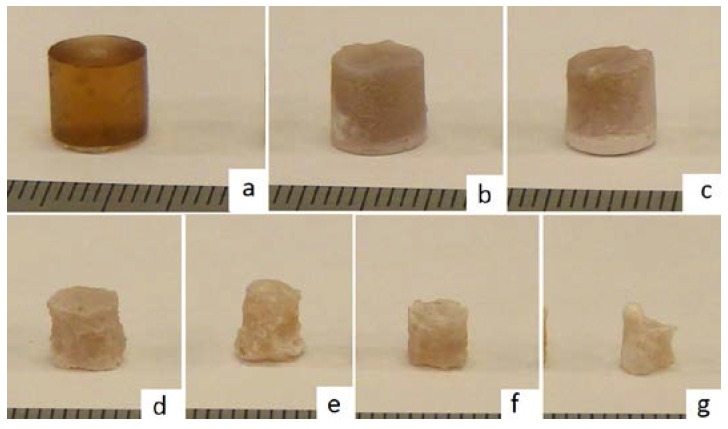
Photographs of PLGA cylinders eroded in 1 M NaOH medium at 55 °C at (**a**) 0 h; (**b**) 12 h; (**c**) 30 h; (**d**) 36 h; (**e**) 42 h; (**f**) 48 h; and (**g**) 54 h.

**Figure 7:**
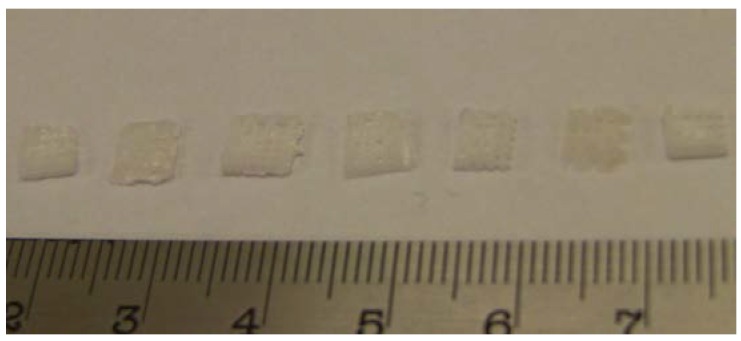
Photograph of PCL scaffolds eroded in 5 M NaOH medium at 55 °C at *t* = 0, 5, 20, 25, 30, 35, and 40 h.

Monitoring of the geometry was employed successfully to measure changes in the diameter of the PCL tissue scaffold layer-by-layer strands, and the diameters of the PLGA cylinders, and to correlate these changes with mass loss in a challenging environment where the samples are surrounded by a medium of similar density and X-ray absorption characteristics. A representative example of the DEI images gained of the scaffolds and implants are shown in Figure 5. Photographs of the PLGA cylinders and PCL scaffolds during degradation are presented in Figure 6 and Figure 7.

**Figure 8:**
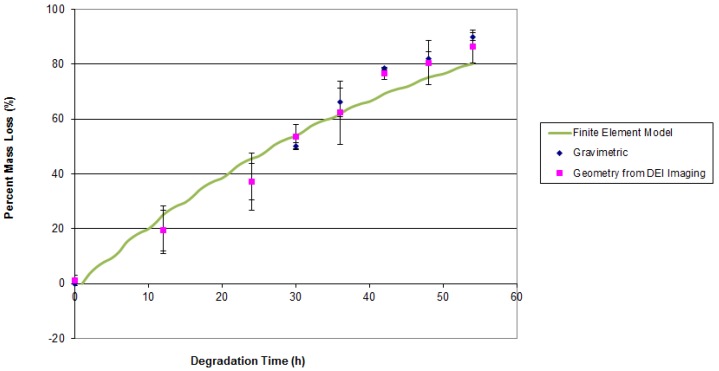
Mass loss for PLGA cylinders characterized by DEI imaging and traditional gravimetric experimental methods.

**Figure 9:**
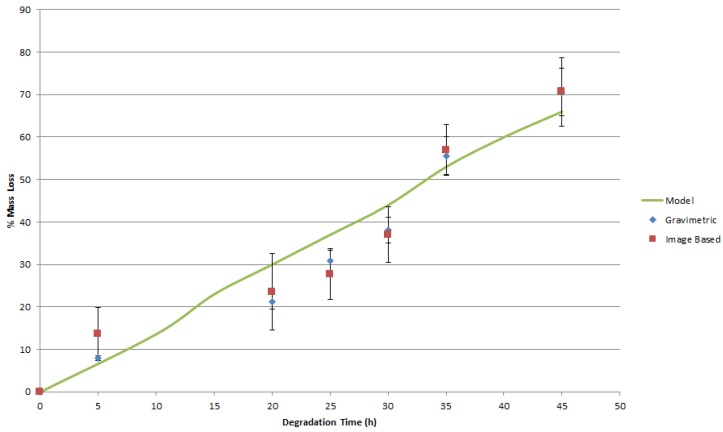
Mass loss for PCL scaffolds characterized by DEI imaging and traditional gravimetric experimental methods for model validation, and the predictions of model when *k*_3_ = 0.28 h^−1^.

In order to estimate mass from the images, the average strand/cylinder diameter in the scaffold/implant was measured. These average diameters were then utilized to estimate the remaining volume of the scaffold/implant, with the known density of PCL or PLGA giving the remaining mass from the image-based time-dependent volume estimate. The experimental data from both this approach and traditional gravimetric analysis were in acceptable agreement, with a percent error of less than 9% observed between the two methods, as illustrated in Figure 8 and Figure 9. The good agreement between estimations of mass loss from image based volume calculations and the physical weighing of the samples indicates that the assumption that the most significant degradation mechanism is surface erosion, and that there are no chemical changes to the bulk scaffold material during degradation is supported. The mass of the scaffold is therefore only a function of the remaining scaffold material volume. It is also implied that the assumption of relatively uniform degradation is reasonable in this particular case where the degrading medium was static and fluid flow effects on degradation were minimal.

**Figure 10:**
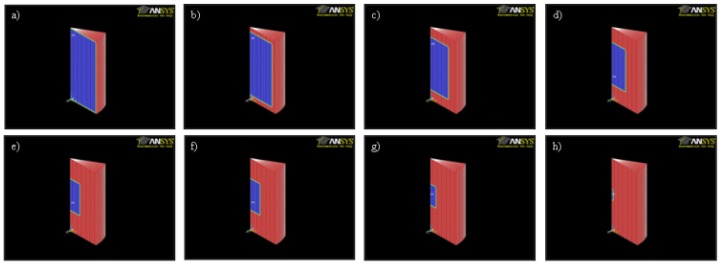
Geometry after (**a**) 1; (**b**) 8; (**c**) 16; (**d**) 24; (**e**) 32; (**f**) 40; (**g**) 48; and (**h**) 60 h of degradation for PLGA cylinders.

**Figure 11:**
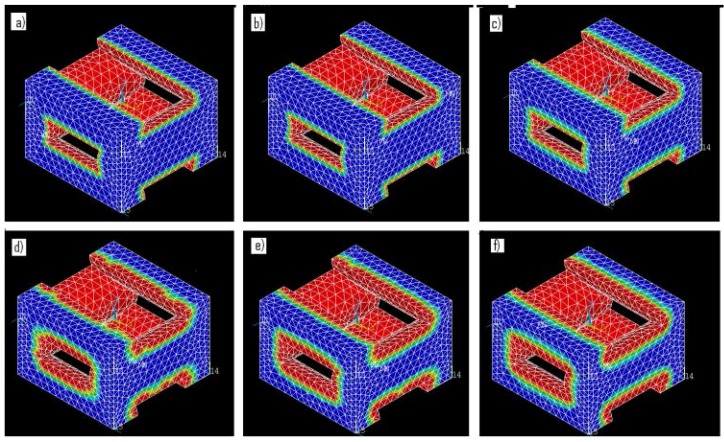
Geometry after (**a**) 1; (**b**) 5; (**c**) 10; (**d**) 15; (**e**) 30; and (**f**) 45 h of degradation for PCL rapid prototyping scaffold samples.

Once experimental data on the mass loss with time was available, the parameters of the finite element (FE) model of mass loss were adjusted using the cyclic coordinate descent algorithm [45] until the best agreement between model predictions and the actual observed behavior was obtained. In brief, this method selects a single free parameter in the model for optimization and calculates the derivative of the objective function with respect to that particular free parameter. The single selected free parameter is then changed to bring the value of the objective function as close to zero as possible, while all of the other free parameters are held at their current value. The algorithm then repeats this process for all of the other identified free parameters. This process is repeated until convergence is achieved. The objective function (5) reached a minimum value of 47.1 for the PLGA implants and a minimum value of 33.3 for the PCL scaffolds. When these minimum values for (5) were obtained, the free parameter k_3_ was set to 3.74 h^−1^ for the PLGA cylinders and 0.28 h^−1^ for the PCL scaffolds. These model parameters are valid for devices manufactured out of PLGA and PCL and degraded in 1 M and 5 M NaOH solutions, respectively, at a temperature of 55 °C. These models with the identified parameters were able to return the observed mass loss in the experimental data. The geometry of the devices as predicted by the mass loss model are presented in Figure 10 and Figure 11, where red regions indicate full absorption, green indicates partial degradation, and blue regions retain their initial molecular weight. The maximum observed difference between model predictions and experiment was 20%. It is apparent in Figure 10 and Figure 11 that the predicted geometry of the devices with time resembles their actual physical appearance, suggesting that the degradation mechanisms present in the physical system are all being successfully represented in the mathematical models of degradation and mass loss. The mathematical model predictions found in Figure 8 also display a gradual decay in the rate of mass loss as degradation proceeds. This behavior is expected, since the interface area of the implant that is in contact with the degradation medium decreases in size as the diameter and height of the implant decreases. The rapid prototyping scaffolds, on the other hand, display essentially linear degradation.

In the work of [46] a cylindrical surface eroding polymer implant 4 mm in length with a diameter of 3 mm composed of poly-trimethylene carbonate (PTMC) is degraded within a rabbit animal model. The observed experimental degradation of the PTMC implant by enzymatic surface degradation may be related to the degradation of PCL in a 5 M solution of NaOH at 55 °C, and the degradation of PLGA in a 1 M solution of NaOH at 55 °C. This allows for the creation of device designs experimentally by employing a representative material/degradation medium that experiences rapid degradation. The behavior of these analogous devices composed of PCL or PLGA can then be employed to estimate the behavior of devices composed of PTMC in vivo. 

The degradation of PCL and PLGA cylindrical implants in NaOH at 55 °C was simulated mathematically with the above estimated values for *k*_3_, and the implant geometry found in [46]. It was found that the 20% mass loss in vivo for PTMC implants after 8 weeks is expected to occur in the analogous accelerated degradation system of PCL/NaOH at 55 °C after 36 h, and after 9 h for PLGA/NaOH at 55 °C. These results suggest that a PLGA or PCL scaffold in NaOH can be employed as an analogous system to PTMC in vivo without the need for an approximately 56 day period to evaluate each potential scaffold design. This experimental approach to scaffold design may be of interest if the scaffold degradation is influenced by both the area of the interface surface and the movement of the degradation medium. If the degradation medium is not static but rather in motion, the current mathematical degradation model is unable to consider the resultant enhancement to the degradation of the scaffold or the possibility of heterogeneous rather than uniform degradation. In this case, an experimental study of the scaffold degradation with an analogous system may allow this effect to be explored for multiple scaffold geometries within a practical degradation time.

**Figure 12:**
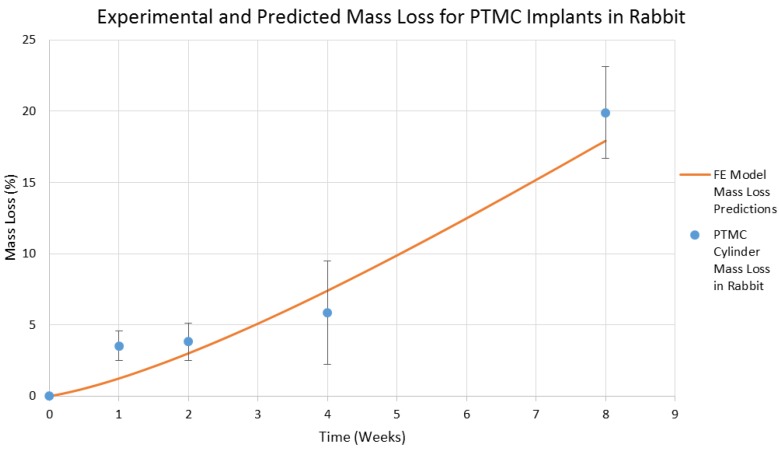
Experimental measurements of mass loss for PTMC cylindrical implant and mass loss model predictions when *k*_3_ = 0.00144 h^−1^.

**Figure 13:**
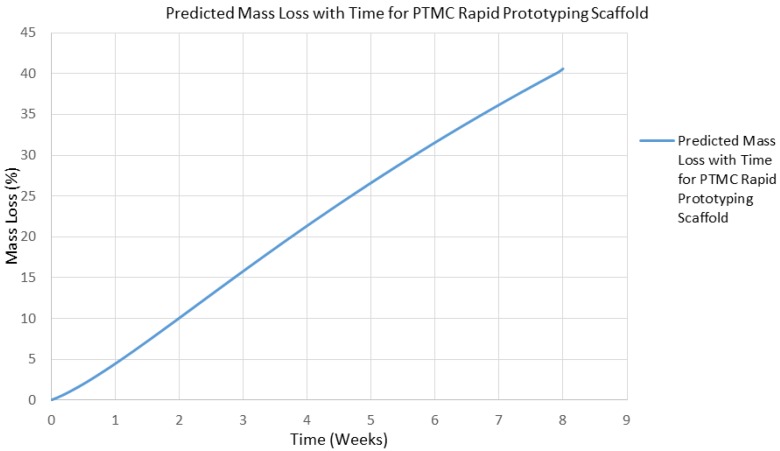
In vivo degradation behavior of poly-trimethylene carbonate (PTMC) rapid prototyping scaffold within rabbit as predicted by mass loss model with *k*_3_ = 0.00144 h^−1^.

Finally, the developed degradation model was fitted to the in vivo experimental data found in [46]. The degradation model was able to successfully represent the mass loss of a PTMC implant when *k*_3_ = 0.00144 h^−1^, as illustrated in Figure 12, with an *R*^2^ value of 0.957. This result demonstrates that the mass loss model developed in this study is able to successfully represent the degradation of a surface eroding polymer in the living environment. This in vivo data was originally obtained using current explanation methods to characterize the in vivo degradation of the implant, but it may also have been obtained remotely in situ using the imaging based approach developed in this study. The model with this value for the free parameter *k*_3_ was then employed to simulate the degradation of a PTMC rapid prototyping scaffold. The model’s estimate for the lifespan of such a scaffold in the living environment is presented in Figure 13. The rapid prototyping scaffold is predicted to degrade almost twice as quickly as the cylindrical implant, which is a reasonable prediction since the size of the interface area of the rapid prototyping scaffold is larger than that of the cylindrical implant.

It is clear from Figure 8 and Figure 9 that the geometry signal of the scaffold is sufficient to remotely infer the mass of the scaffold without having to explant the device, as is the traditional method for mass loss monitoring. Furthermore, there is no need to add substances or tracers, radioactive or otherwise, to the scaffold to track the movement of mass in the body, as is necessary for some of the other approaches to monitoring mass loss in vivo [47,48]. However, since the volume of an object is proportional to its dimensions to the third power, any error in the measurement of the geometric parameters of the scaffold will be magnified in the prediction of mass from geometry measurements. The resolution of the imaging system must therefore be sufficient to achieve an acceptable error in the estimation of scaffold/implant mass. The existing imaging system was capable of estimating mass within 25% of the actual mass as characterized by gravimetric methods. The way is now clear to begin applying this approach to examining the in vivo surface erosion of devices and comparing these observed results to those obtained in simple analogs to the living environment such as phosphate buffered saline or cell culture medium immersions.

## 5. Conclusions and Future Work

DEI was successfully employed to visualize PLGA and PCL scaffolds in an imaging environment similar to that of a living body, where both the density of the surroundings and implant are similar. Monitoring of the geometry with time allowed the volume and hence the mass of the scaffolds to be estimated as a function of time. These estimates of mass agreed reasonably with the directly measured mass loss. This data was then able to allow the estimation of the parameters in a mass loss mathematical model. In addition, the degradation of PLGA and PCL at a specific medium temperature and pH is similar to the degradation of PTMC in the living environment of a rabbit, with the exception that the accelerated degradation of PLGA and PCL in 1 M and 5 M solutions of NaOH at 55 °C degrade 149 and 37 times faster, respectively. Future work will include the application of this image-based monitoring method to implant structures that display bulk degradation, where the initial formation of interior voids will be the critical state employed to estimate model parameters. In addition, the next stage in the validation of this approach for mass loss characterization is the evaluation of a PTMC rapid prototyping fabricated scaffold in vivo with the same imaging methodology outlined in this study. Also, the use of leave-one-out-cross-validation for parameter estimation, with an averaging of the estimations for the parameter *k*_3_, and the use of a smaller step-size may improve agreement between the FE models and experiment.

## References

[1] Little C.J., Bawolin N.K., Chen X.B. (2011). Mechanical Properties of Natural Cartilage and Tissue Engineered Constructs. Tissue Eng. Part B.

[2] Bawolin N.K., Zhang W.J., Chen X.B. (2010). A Brief Review of the Modelling of the Time Dependent Mechanical Properties of Tissue Engineering Scaffolds. J. Biomimetics Biomater. Tissue Eng..

[3] Woodruff M.A., Hutmacher D.W. (2010). The return of a forgotten polymer: Polycaprolactone in the 21st century. Prog. Polym. Sci..

[4] Zhuang H., Han Y., Feng A. (2008). Preparation, mechanical properties and in vitro biodegradation of porous magnesium scaffolds. Mater. Sci. Eng. C.

[5] Ibim S.E., Uhrich K.E., Attawia M., Shastri V.R., El-Amin S.F., Bronson R., Langer R., Laurencin C.T. (1998). Preliminary in vivo report on the osteocompatibility of poly(anhydride-co-imides) evaluated in a tibial model. J. Biomed. Mater. Res..

[6] Ibim S.E., Uhrich K.E., Bronson R., El-Amin S.F., Langer R.S., Laurencin C.T. (1998). Poly(anhydride-co-imides): In vivo biocompatibility in a rat model. Biomaterials.

[7] Lam C.X.F., Hutmacher D.W., Schantz J.T., Woodruff M.A., Teoh S.H. (2009). Evaluation of polycaprolactone scaffold for 6 months in vitro and in vivo. J. Biomed. Mater. Res. Part A.

[8] Holy C.E., Dang S.M., Davies J.E., Shoichet M.S. (1999). In vitro degradation of a novel poly(lactide-co-glycolide) 75/25 foam. Biomaterials.

[9] Gough J.E., Christian P., Unsworth J., Evans M.P., Scotchford C.A., Jones I.A. (2004). Controlled degradation and macrophage responses of a fluoride-treated polycaprolactone. J. Biomed. Mater. Res. A.

[10] Arosio P., Busini V., Perale G., Moscatelli D., Masi M. (2008). A new model of resorbable device degradation and drug release—Part I: Zero order model. Polym. Int..

[11] Pena J., Corrales T., Izquierdo-Barba I., Doadrio A.L., Vallet-Regi M. (2006). Long term degradation of poly(3-caprolactone) films in biologically related fluids. Polym. Degrad. Stab..

[12] Pena J., Corrales T., Izquierdo-Barba I., Concepcion Serrano M., Teresa Portoles M., Pagani R., Vallet-Regi M. (2006). Alkaline-treated poly(ɛ-caprolactone) films: Degradation in the presence or absence of fibroblasts. J. Biomed. Mater. Res. Part A.

[13] Tay F.R., Pashley D.H., Yiu C.K.Y., Yau J.Y.Y., Yiu-fai M., Loushine R.J., Norman Weller R., Frank Kimbrough W., King N.M. (2005). Susceptibility of a Polycaprolactone-Based Root Canal Filling Material to Degradation. II. Gravimetric Evaluation of Enzymatic Hydrolysis. J. Endod..

[14] Stevens M.M., Marini R.P., Schaefer D., Aronson J., Langer R., Prasad Shastri V. (2005). In vivo engineering of organs: The bone bioreactor. PNAS.

[15] Lu L., Peter S.J., Lyman M.D., Lai H.-L., Leite S.M., Tamada J.A., Uyama S., Vacanti J.P., Langer R., Mikos A.G. (2000). In vitro and in vivo degradation of porous poly(DL-lactic-co-glycolic acid) foams. Biomaterials.

[16] Pitt C.G., Gratzl M.M., Kimmel G.L., Surleas J., Schindler A. (1981). Aliphatic polyesters II: The degradation of poly (DL-lactide), poly (ε-caprolactone) and their copolymers in vivo. Biomaterials.

[17] Doyle C., Tanner E.T., Bonfiel W. (1991). In vitro and in vivo evaluation of poly-hydroxy-butyrate and of poly-hydroxy-butyrate reinforced with hydroxy-apatite. Biomaterials.

[18] Pistner H., Stallforth H., Gutwald R., Muhling J., Reuther J., Michel C. (1994). Poly(L-lactide): A long-term degradation study in vivo Part II: Physico-mechanical behavior of implants. Biomaterials.

[19] Ho S.T., Hutmacher D.W. (2006). A comparison of micro CT with other techniques used in the characterization of scaffolds. Biomaterials.

[20] Ciocca L., Lesci I.G., Mezini O., Parrilli A., Ragazzini S., Rinnovati R., Romagnoli N., Roveri N., Scotti R. (2015). Customized hybrid biomimetic hydroxyapatite scaffold for bone tissue regeneration. J. Biomed. Mater. Res. Part B Appl. Biomater..

[21] Wong H.M., Chub P.K., Leung F.K.L., Cheung K.M.C., Luka K.D.K., Yeung K.W.K. (2014). Engineered polycaprolactone–magnesium hybrid biodegradable porous scaffold for bone tissue engineering. Prog. Nat. Sci. Mater. Int..

[22] Klodowski K., Kaminski J., Nowickab K., Tarasiuk J., Wronski S., Swietek M., Blazewiczb M., Figiel H., Tureka K., Szponder T. (2014). Micro-imaging of implanted scaffolds using combined MRI and micro-CT. Comput. Med. Imaging Graphics.

[23] Wagner D.W., Lindsey D.P., Beaupre G.S. (2011). Deriving tissue density and elastic modulus from microCT bone scans. Bone.

[24] Bawolin N., Dolovich A.T., Zheng W.J., Chen X.B. (2015). Characterization of Mechanical Properties of Tissue Scaffolds by Phase Contrast Imaging and Finite Element Modeling. ASME J. Biomech. Eng..

[25] Izadifar Z., Honaramooz A., Wiebe S., Belev G., Chen X.B., Chapman D. (2015). Low-Dose Phase-based X-ray Imaging Techniques for in situ Soft Tissue Engineering Assessments. Biomaterials.

[26] Izadifar Z., Chapman L.D., Chen X.B. (2013). Computer Tomography Diffraction Enhanced Imaging for in situ Visualization of Tissue Scaffolds Implanted in Cartilage. Tissue Eng. Part C.

[27] Olubamiji A.D., Izadifar Z., Zhu N., Chang T.J., Chen X.B., Eames B.F. (2016). Using SR-inline-PCI-CT to visualize 3D-printed hybrid constructs for cartilage tissue engineering. J. Synchrotron Radiat..

[28] Zhu N., Chapman D., Cooper D., Schreyer D., Chen X.B. (2011). X-ray Diffraction Enhanced Imaging as a Novel Method to Visualize Low Density Scaffolds in Soft Tissue Engineering. Tissue Eng. Part C.

[29] Olubamiji A., Izadifar Z., Chen X.B. (2014). Synchrotron imaging techniques for bone and cartilage tissue engineering: Potentials, current trends, and future directions. Tissue Eng. Part C.

[30] Chapman D., Thomlinson W., Johnston R.E., Washburn D., Pisano E., Gmür N., Zhong Z., Menk R., Arfelli F., Sayers D. (1997). Diffraction Enhanced x-ray Imaging. Phys. Med. Biol..

[31] Dierker J., Joite-Barfub S., Sabel M., Aichinger H. (2012). Radiation Exposure and Image Quality in X-ray Diagnostic Radiology: Physical Principles and Clinical Applications.

[32] Brankov J.G., Saiz-Herrandez A., Wernick M.N. Noise analysis for diffraction enhanced imaging. Proceedings of the 2004 IEEE International Symposium on Biomedical Imaging: From Nano to Macro.

[33] Muehleman C., Li J., Connor D., Parham C., Pisano E., Zhong Z. (2009). Diffraction-Enhanced Imaging of Musculoskeletal Tissues Using a Conventional X-ray Tube. Acad. Radiol..

[34] Engineer C., Parikh J., Raval A. (2011). Review on Hydrolytic Degradation Behavior of Biodegradable Polymers from Controlled Drug Delivery System. Trends Biomater. Artif. Organs..

[35] Webb M.A., Belev G., Wysokinski T.W., Chapman D. Diffraction enhanced imaging computed tomography (DEI-CT) at the BMIT facility at the Canadian Light Source. Proceedings of the 7th Medical Applications of Synchrotron Radiation Workshop (MASR 2012) Shanghai Synchrotron Radiation Facility (SSRF).

[36] Ang K.C., Leong K.F., Chua C.K., Chandrasekaran M. (2007). Compressive properties and degradability of poly(e-caprolatone)/hydroxyapatite composites under accelerated hydrolytic degradation. J. Biomed. Mater. Res. A.

[37] Lam C.X.F., Savalani M.M., Teoh S.-H., Hutmacher D.W. (2008). Dynamics of in vitro polymer degradation of polycaprolactone-based scaffolds: Accelerated versus simulated physiological conditions. Biomed. Mater..

[38] Bawolin N.K. (2013). Synchrotron Based Imaging for Mass Loss Characterization.

[39] Guo Q., Groeninckx G. (2001). Crystallization Kinetics Poly(e-caprolactone) in Miscible Thermosetting Polymer Blends of Epoxy Resin and Poly (e-caprolactone). Polymer.

[40] Wang Y., Pan J., Han X., Sinka C., Ding L. (2008). A Phenomenological Model for the Degradation of Biodegradable Polymers. Biomaterials.

[41] Bawolin N.K., Li M.G., Chen X.B., Zhang W.J. (2010). Modeling Material-Degradation-Induced Elastic Property of Tissue Engineering Scaffolds. J. Biomech. Eng..

[42] Husmann M., Schenderlein S., Luck M., Lindner H., Kleinebudde P. (2002). Polymer erosion in PLGA microparticles produced by phase separation method. Int. J. Pharm..

[43] Wang S., Lu L., Yaszemski M.J. (2006). Bone Tissue-Engineering Material Poly(propylene fumarate): Correlation between Molecular Weight, Chain Dimensions, and Physical Properties. Biomacromolecules.

[44] Han X., Pan J. (2009). A model for simultaneous crystallisation and biodegradation of biodegradable polymers. Biomaterials.

[45] Bezdek J.C., Hathaway R.J., Pal N.R., Sugeno M. (2002). Some Notes on Alternating Optimization. Advances in Soft Computing, Vol. 2275 of Lecture Notes in Artificial Intelligence.

[46] Zhang Z., Kuijer R., Bulstra S.K., Grijpma D.W., Feijen J. (2006). The in vivo and in vitro degradation behavior of poly(trimethylene carbonate). Biomaterials.

[47] Sun H., Mei L., Song C., Cui X., Wang P. (2006). The in vivo degradation, absorption and excretion of PCL-based implant. Biomaterials.

[48] Artzi1 N., Oliva N., Puron C., Shitreet S., Artzi S., Ramos A., Groothuis A., Sahagian G., Edelman E. (2011). In vivo and in vitro tracking of erosion in biodegradable materials using non-invasive fluorescence imaging. Nat. Mater..

